# The Life and Fate of Mesenchymal Stem Cells

**DOI:** 10.3389/fimmu.2014.00148

**Published:** 2014-05-19

**Authors:** Elke Eggenhofer, Franka Luk, Marc H. Dahlke, Martin J. Hoogduijn

**Affiliations:** ^1^Department of Surgery, University Medical Center Regensburg, Regensburg, Germany; ^2^Nephrology and Transplantation, Department of Internal Medicine, Erasmus Medical Center, Rotterdam, Netherlands

**Keywords:** mesenchymal stem cells, culture, migration, homing, survival

## Abstract

Mesenchymal stem cells (MSC) are present throughout the body and are thought to play a role in tissue regeneration and control of inflammation. MSC can be easily expanded in vitro and their potential as a therapeutic option for degenerative and inflammatory disease is therefore intensively investigated. Whilst it was initially thought that MSC would replace dysfunctional cells and migrate to sites of injury to interact with inflammatory cells, experimental evidence indicates that the majority of administered MSC get trapped in capillary networks and have a short life span. In this review, we discuss current knowledge on the migratory properties of endogenous and exogenous MSC and confer on how culture-induced modifications of MSC may affect these properties. Finally, we will discuss how, despite their limited survival, administered MSC can bring about their therapeutic effects.

## Introduction

Mesenchymal stem cells (MSC) are present in virtually all tissues where they interact with tissue cells and, under conditions of inflammation, with immune cells. They have multipotent differentiation capacity, which allows them to differentiate in osteoblasts, adipocytes, chondrocytes, and other cell types and they have immunomodulatory capacity by sensing and controlling inflammation and modifying the proliferation and cytokine production of lymphocytes and myeloid-derived immune cells (1). MSC are relatively easy to isolate and expand in culture and therefore have prospect as a therapeutic tool in degenerative and autoimmune disease and in transplantation. Experimental pre-clinical and clinical studies with MSC are based on the isolation of MSC from bone marrow or adipose tissues and the expansion of MSC *in vitro*. Culture-expanded MSC can be administered via different routes, including via intramuscular injections, subcutaneously and via intravenous infusion. While it was originally anticipated that MSC could be used for replacement of dysfunctional cells via their capacity to differentiate into tissue cells (2), the current paradigm is that MSC support resident progenitor cells via paracrine mechanisms (3, 4). Even this idea may need refinement as recent studies suggest that MSC may not have a long lifespan after administration (5, 6). The reason why this may be the case is not clear. A rapid disappearance of MSC raises the question of how MSC therapy might work. It is possible that a small fraction of administered MSC escapes death and migrates to sites of injury and inflammation and that these MSC bring about the beneficial effects of MSC. Another possibility is that MSC are able to rapidly pass on their effect to other cells that subsequently mediate tissue repair or immunomodulation. Even the clean-up process of MSC itself may be a trigger for the therapeutic effects of MSC. In the present review, we discuss recent findings on the life and fate of tissue resident and administered MSC and try to link these findings with the regenerative and immunomodulatory effects of MSC.

## Migration of MSC *in vitro*

It has been shown that MSC migrate in response to many chemotactic factors, like platelet-derived growth factor-AB (PDGF-AB), insulin-like growth factor-1 (IGF-1), the chemokines RANTES, macrophage-derived chemokine (MDC), and stromal-derived factor-1 (SDF-1) (7). Accordingly, MSC express the tyrosine kinase receptors for PDGF and IGF, as well as the RANTES and MDC receptors CCR2, CCR3, and CCR4, and the SDF-1 receptor CXCR4. Most chemokines are more effective on TNFα-primed cells, suggesting that the mobilization of MSC and their subsequent homing to injured tissues may depend on the systemic and local inflammatory state (7). Whereas culture-expanded MSC are widely used in clinical trials, it is not clear how cultivation and GMP manufacturing processes may influence the homing properties of MSC *in vivo*. However, it has been shown that cell culture duration and the degree of cell expansion has a clear impact on MSC morphology, differentiation, viability, and migratory properties (8). Furthermore, several groups have shown that freshly isolated MSC show superior homing ability compared to expanded MSC (9, 10) and that different MSC subtypes, like classical MSC and multipotent adult progenitor cells have different migration potential (11) in *in vitro* migration assays. This suggests that different MSC preparations show variation with regard to their homing receptor expression, and this may result in differences in their therapeutic effect. Culture conditions appear therefore a potent tool in modulating the effect of MSC administration.

KEY CONCEPT 1. Migration of MSC MSC express a range of chemokine receptors, allowing them to specifically migrate towards chemokine gradients.

## Migration of Endogenous MSC

The study of endogenous MSC migration *in vivo* is complex. MSC are located in the bone marrow, from where they may migrate to other sites via mechanisms potentially similar to those exploited by hematopoietic stem cells. However, MSC may be resident in peripheral blood, which would make the specific identification of migrating MSC difficult. The detection of MSC in the circulation is controversial. While some studies showed that cord blood and mobilized peripheral blood are enriched for MSC (12), others failed to detect MSC in the circulation (13), even after stem cell mobilization or in cord blood (14). MSC have been detected in peripheral blood in patients with hip bone fractures (15), but it can be questioned whether MSC are present in the blood of these patients via active recruitment or whether mechanical disruption of the bone tissue is responsible for this. A study in rat showed that hypoxia induced the mobilization of MSC in peripheral blood (16) and a murine study investigating the mobilization of fluorescent MSC injected in the bone marrow of non-fluorescent animals detected an increased number of fluorescent MSC in the blood after the induction of liver damage (17), suggesting that systemic signals trigger the release of MSC from the bone marrow.

KEY CONCEPT 2. Migration of endogenous MSC The migration of endogenous MSC is controversial. Hard evidence for the migration of MSC via the bloodstream is sparse. As MSC are present in virtually all tissues, they may migrate within tissues to sites of injury or inflammation.

**Figure 1:**
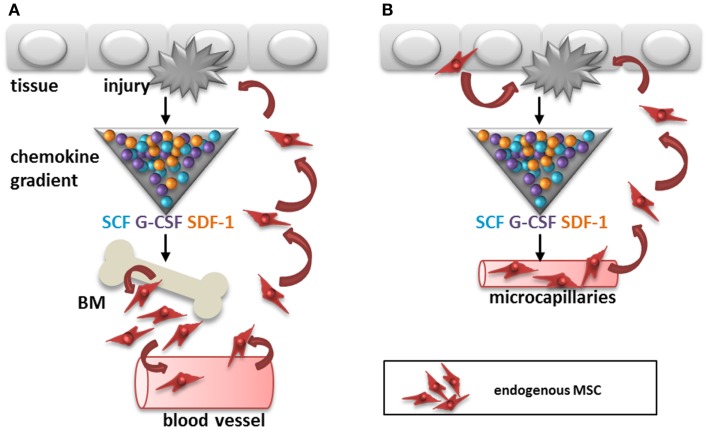
**Two potential routes for recruitment of endogenous MSC after tissue injury**. **(A)** Cytokines, chemokines, and growth factors like SCF, G-CSF, and SDF-1 are released by the injured tissue, which may trigger recruitment of MSC from the bone marrow to sites of injury via the circulation. **(B)** Alternatively, MSC are recruited from within tissues to sites of injury via migration within the stroma or via micro-capillaries. SCF, stem cell factor; G-CSF, granulocyte-colony stimulating factor; SDF-1, stromal cell derived factor-1; BM, bone marrow.

One of the difficulties detecting circulating MSC is that their immunophenotype is not necessarily the same as that of culture-expanded MSC. Several of the markers used for the identification of MSC have an adhesion function and their expression is therefore likely to be different on non-adherent MSC. Furthermore, although the first compartment to search for circulating cells is usually the blood stream, a recent report suggests that adipose tissue derived MSC may migrate via the lymphatic system (18). It was demonstrated that MSC can be released by adipose tissue or bone marrow in response to inflammation and that they accumulate in lymph nodes and blood vessels via CXCL12 (SDF-1)/CXCR4 dependent mechanisms (Figure 1A). Finally, one may wonder whether the recruitment of MSC from distant sites is required for the control of immune responses and initiation of repair in tissues as MSC are found locally in all tissues, from skin to brain (13). In case of injury, local MSC from tissue or blood vessels need to travel only short distances to get to sites of injury and thereby cut the blood stream route short (Figure 1B).

Transplanted organs provide a unique opportunity for studying the migration of MSC, as MSC can be cultured from tissue biopsies and their donor or recipient origin determined by HLA typing. A study in heart transplant patients demonstrated that MSC present in transplanted hearts were all of donor origin (19). No MSC of recipient origin were found, even not many years after transplantation. Similar data were found in lung transplant patients (20). These data suggest that MSC do not migrate between tissues, not even under inflammatory conditions as found in transplanted organs. At the same time these data demonstrate that MSC can in principle survive after transplantation. A major difference, however, between these studies and studies that show that **MSC are short-lived after administration** (5) as discussed in the next paragraph, is that MSC that are administered as a form of therapy are culture-expanded and administered without supporting tissue around them, as is the case in organ transplantation. The localization and microenvironment that MSC face after transplantation as a cell suspension or embedded in an organ are not comparable and are likely to be responsible for the apparent discrepant survival data.

KEY CONCEPT 3. MSC are short-lived after administration *In vitro* expanded MSC have a short lifetime after *in vivo* administration. Intravenously infused MSC home to the lungs, from where they disappear within 24h. Living MSC are not detected at other tissue sites.

## Localization and Homing of Exogenous MSC

The use of MSC for immunomodulation or regenerative therapy in pre-clinical models for autoimmune disease, transplantation, or degenerative disease offers the possibility of studying the migration of exogenous MSC. Several studies have demonstrated the homing of administered MSC to sites of injury (21–23). The migration of MSC to such sites may be dependent on chemotactic signals derived from injured or inflamed tissues. A study using a model of acute kidney injury demonstrated that the migration of MSC to the injured kidney is dependent on CD44 expression on MSC (24). The expression of the chemokine receptor CXCR4 on MSC also appears to play a role, as overexpression of CXCR4 increases the homing of MSC to injured kidneys (25).

**Figure 2:**
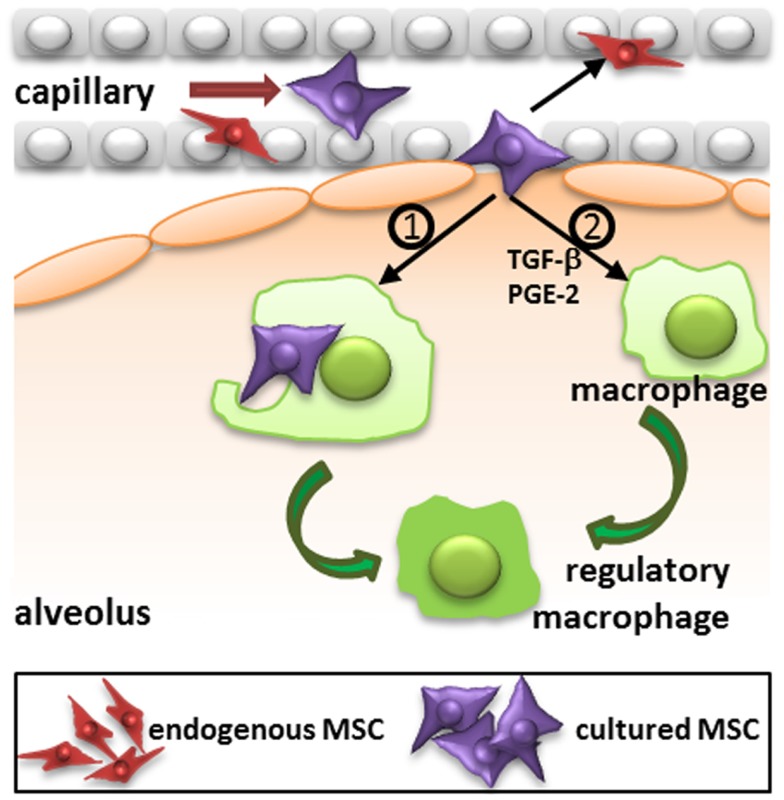
**Proposed mechanism of action of intravenously injected cultured MSC**. Administered culture-expanded MSC (large violet cells) target immune cells, including macrophages, and resident progenitor cells, including MSC (small red cells) via their secretome (e.g., TGF-β, PGE-2, and other factors) to stimulate immunomodulatory and regenerative processes. In addition, phagocytosis of MSC by macrophages may induce the formation of regulatory macrophages. TGF-β, transforming growth factor-β; PGE-2, prostaglandin E2.

The migration of MSC is likely to depend on their route of administration. Most studies use the intravenous route and it has become clear that a large proportion of MSC that are injected via this route are trapped in the lungs upon first passage (5, 10, 23, 26). After 24 h MSC are relocated to other organs, in particular the liver and also the spleen (5, 27). MSC also reappear at injured tissue sites (27). It is however questionable whether MSC that leave the lungs are still viable. In our previous work, we demonstrated that MSC are no longer viable 24 h after intravenous infusion, but that their radioactive label was still detectable in the liver (5). The elimination of MSC may be dependent of immunological mechanisms, which will be discussed below. The potential rapid disappearance of infused MSC does not rule out a functional effect of the cells. It has for instance been demonstrated that the phagocytosis of dead MSC induces the generation of macrophages with a regulatory phenotype (28) (Figure 2). It is also possible that a small proportion of cells escapes elimination and is responsible for the therapeutic effects of MSC.

Evasion of the lung trap may improve the survival of MSC and may affect the distribution of the cells after administration. Arterial injection may ensure better delivery of MSC to organs of interest. MSC injected in the renal arteries are retained in the glomeruli for at least several days (29). A study examining the engraftment of MSC in the liver concluded that administration via the portal vein led to far better engraftment than administration via the vena cava (30). While intravenous infusion of MSC for treatment of cardiac infarct has been shown long time ago to lead to poor engraftment in the ischemic heart, injection of MSC directly in the myocardium improves the uptake of cells (26, 31).

It thus appears that MSC are trapped in the first micro capillary network they encounter. The question is why this is the case and whether the entrapment of MSC in such networks perhaps contributes to in particular the immunomodulatory effects of MSC treatment?

## Effect of Culture on the Migration and Survival of MSC

Mesenchymal stem cells cultures are routinely obtained by adherence of MSC to plastic culture flasks and propagation of the cells in medium containing 10–15% fetal calf serum (32). However, **culture affects the phenotype of MSC**. Various studies show that surface markers of freshly isolated MSC differ from those of cultured MSCs. It has for instance been described that adipose tissue derived MSC are contained in the CD34^+^ cell fraction of the stromal vascular fraction, but CD34 expression is lost during MSC cell expansion in culture (33). Recently, Braun et al. (34) showed that upon activation in culture, MSC upregulate CD105, CD146, and CD271 and propose that this upregulation is required for improved motility and attachment to the plastic. In accordance with this hypothesis, we as well as other groups have observed a change in MSC morphology during early culture phase. Over time, small round-shaped cells change into large spindle shaped cells (34, 35) and cultured MSC become around 20 μm in diameter (36, 37). Since this diameter is larger than the size of pulmonary micro-capillaries, it is not surprising that after intravenous infusion of MSC the majority of the cells are trapped in the lungs (5, 37). Furthermore, the modulated expression of cell adhesion and chemoattraction molecules on cultured MSC may account for a different migratory behavior of cultured MSC compared to non-cultured MSC. The shortevity of MSC after infusion may also have a relation with the culture-induced changes in MSC phenotype. We and others have demonstrated that activated NK cells can lyse culture-expanded MSC of not only allogeneic but also autologous origin (38, 39), suggesting that culture may induce changes in MSC that makes them targets for NK cells. Cultured MSC may also appear foreign for macrophages, and trigger a clean-up response after administration, which may result in an immunosuppressive effect as discussed above (28). These data indicate that it is not unlikely that at least some of the effects observed upon administration of MSC depend on MSC properties acquired during culture expansion.

KEY CONCEPT 4. Culture affects the phenotype of MSC Culture medium and plastic adherence have a major impact on the phenotype of MSC. The size of MSC dramatically increases in culture and the expression of adhesion molecules is strongly up regulated. This affects the distribution of MSC after administration.

## How Does MSC Therapy Work?

**Figure 3:**
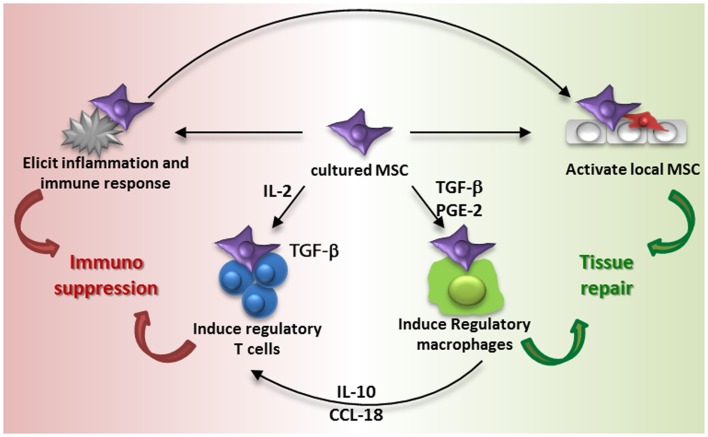
**Proposed model of MSC contribution to immune suppression and tissue repair**. Intravenous administration of culture-expanded MSC (violet cells) leads to modulation of the function of endogenous MSC (red cells) and macrophages via the secretome of the administered cells (e.g., TGF-β, PGE-2, and other factors) and phagocytosis of MSC by macrophages. The activation of resident MSC and induction of regulatory macrophages induces tissue regeneration. MSC furthermore induce regulatory T cells via different mechanisms, including the secretion of TGF-β and an indirect elevation of IL2. Administration of MSC also elicits a systemic immune response, which triggers an immunosuppressive response.

Series of observations on the migration, distribution, and survival of MSC after administration contrast with the original ideas on the life and fate of MSC and make it hard to understand how MSC therapy works. In the 1960s, cells with osteogenic differentiation capacity were identified in the bone marrow by Friedenstein and colleagues (40). Later it was shown that in addition to osteogenic differentiation, these cells, MSC, were able to undergo adipogenic, chondrogenic, and myogenic differentiation (41). As a logic consequence it was believed that MSC could be expanded in culture, administered and that they would engraft and differentiate into functional cells. Some of the early trials with MSC that took place in osteogenesis imperfecta patients were based on this idea (42). It turned out, however, that culture-expanded MSC show little engraftment and it is proposed that the secretion of trophic factors and immunomodulatory cytokines and chemokines by MSC are at the base of the therapeutic effects (43, 44). However, the accumulation of MSC in the lungs after intravenous infusion, their short survival time and limited distribution to other sites suggests that MSC rapidly pass on their effect to resident cells, which may subsequently mediate the immunomodulatory and regenerative effect induced by MSC administration. This idea is summarized in Figure 3.

Regulatory T cells are one of the cell types that may bring the effects of MSC forward. MSC have been demonstrated to be able to induce regulatory T cells (45, 46). The mechanisms of MSC-induced regulatory T cell induction depend on local conditions. MSC constitutively secrete TGF-β, which acts as a growth factor for regulatory T cells (45), and under inflammatory conditions MSC express IDO, which plays a role in the generation of regulatory T cells (47). MSC induce *de novo* regulatory T cells by increasing levels of IL2, which occurs when activated T cells are inhibited by MSC in their proliferation but not in their activation (46). Furthermore, monocytes are identified as a crucial intermediate cell type after MSC-induced differentiation in type 2 macrophages. Type 2 macrophages produce IL10 and CCL18, which trigger the induction of regulatory T cells. Type 2 macrophages can furthermore be induced by prostaglandin E2 secretion by MSC (48) and themselves play a role in resolving inflammation and mediating tissue repair (49). It was recently demonstrated that the reparative effect of MSC injected in infarcted myocardium was partially mediated via type 2 macrophages as depletion of these macrophages attenuated the MSC effect (50).

As infused MSC are around only for a short time, their induction of cells with regulatory phenotypes via the secretion of soluble factors has to occur hastily. It is also possible that MSC trigger responses by other cells merely by their presence. We recently demonstrated that **MSC induce a systemic inflammatory response** within hours after infusion (51). Whether this response triggers the induction of regulatory cells and whether it originates from MSC or from resident tissue and/or immune cells remains to be identified. It however indicates that infusion of MSC leads to rapid responses that may subsequently affect other cells. The systemic nature of the response indicates that even though MSC are localized in the lungs after infusion, their effects can reach other body sites, and this may underlie the **mechanism of MSC therapy**. What happens to MSC after they disappear from the lungs is not clear. It may be that small numbers of MSC escape elimination and migrate to sites of injury, but whether these surviving MSC mediate the therapeutic effect or whether the bulk dead cells are responsible for this we cannot conclude about at this moment. There is evidence that dead MSC reappear in the liver (5), and interestingly, macrophages that phagocytized dead MSC have been shown to obtain a regulatory function (28).

KEY CONCEPT 5. MSC induce a systemic inflammatory response MSC induce an inflammatory response after infusion, which is detectable in lung tissue and systemically 2h after administration. This response may lead to the immunomodulatory effects seen after MSC infusion.

KEY CONCEPT 6. Mechanism of MSC therapy The mechanisms of MSC therapy are poorly understood. As MSC have a short survival time after infusion, it is believed that MSC pass on their effects to other cell types. MSC can induce regulatory T cells and regulatory macrophages, which carry on the immunomodulatory and regenerative effects of MSC.

Taking all published data into considering, we have to acknowledge that we yet know little about how MSC therapy works. Further studies aimed at exploring MSC fate and functional properties after administration will have to shine light on this and allow the design of effective MSC therapy.

## Conflict of Interest Statement

The authors declare that the research was conducted in the absence of any commercial or financial relationships that could be construed as a potential conflict of interest.

## References

[1] Bernardo ME Fibbe WE Mesenchymal stromal cells: sensors and switchers of inflammation. Cell Stem Cell (2013) 13(4):392–402 10.1016/j.stem.2013.09.006 24094322

[2] Prockop DJ Marrow stromal cells as stem cells for nonhematopoietic tissues. Science (1997) 276(5309):71–4 10.1126/science.276.5309.71 9082988

[3] Togel F Weiss K Yang Y Hu Z Zhang P Westenfelder C Vasculotropic, paracrine actions of infused mesenchymal stem cells are important to the recovery from acute kidney injury. Am J Physiol Renal Physiol (2007) 292(5):F1626–35 10.1152/ajprenal.00339.2006 17213465

[4] Hocking AM Gibran NS Mesenchymal stem cells: paracrine signaling and differentiation during cutaneous wound repair. Exp Cell Res (2010) 316(14):2213–9 10.1016/j.yexcr.2010.05.009 20471978 PMC2902653

[5] Eggenhofer E Benseler V Kroemer A Popp FC Geissler EK Schlitt HJ Mesenchymal stem cells are short-lived and do not migrate beyond the lungs after intravenous infusion. Front Immunol (2012) 3:297 10.3389/fimmu.2012.00297 23056000 PMC3458305

[6] Liu XB Chen H Chen HQ Zhu MF Hu XY Wang YP Angiopoietin-1 preconditioning enhances survival and functional recovery of mesenchymal stem cell transplantation. J Zhejiang Univ Sci B (2012) 13(8):616–23 10.1631/jzus.B1201004 22843181 PMC3411094

[7] Ponte AL Marais E Gallay N Langonne A Delorme B Herault O The in vitro migration capacity of human bone marrow mesenchymal stem cells: comparison of chemokine and growth factor chemotactic activities. Stem Cells (2007) 25(7):1737–45 10.1634/stemcells.2007-0054 17395768

[8] Wagner W Ho AD Zenke M Different facets of aging in human mesenchymal stem cells. Tissue Eng Part B Rev (2010) 16(4):445–53 10.1089/ten.TEB.2009.0825 20196648

[9] Rombouts WJ Ploemacher RE Primary murine MSC show highly efficient homing to the bone marrow but lose homing ability following culture. Leukemia (2003) 17(1):160–70 10.1038/sj.leu.2402763 12529674

[10] Fischer UM Harting MT Jimenez F Monzon-Posadas WO Xue H Savitz SI Pulmonary passage is a major obstacle for intravenous stem cell delivery: the pulmonary first-pass effect. Stem Cells Dev (2009) 18(5):683–92 10.1089/scd.2008.0253 19099374 PMC3190292

[11] Eggenhofer E Popp FC Mendicino M Silber P Van’t Hof W Renner P Heart grafts tolerized through third-party multipotent adult progenitor cells can be retransplanted to secondary hosts with no immunosuppression. Stem Cells Transl Med (2013) 2(8):595–606 10.5966/sctm.2012-0166 23836805 PMC3726139

[12] Tondreau T Meuleman N Delforge A Dejeneffe M Leroy R Massy M Mesenchymal stem cells derived from CD133-positive cells in mobilized peripheral blood and cord blood: proliferation, Oct4 expression, and plasticity. Stem Cells (2005) 23(8):1105–12 10.1634/stemcells.2004-0330 15955825

[13] da Silva Meirelles L Chagastelles PC Nardi NB Mesenchymal stem cells reside in virtually all post-natal organs and tissues. J Cell Sci (2006) 119(Pt 11):2204–13 10.1242/jcs.02932 16684817

[14] Wexler SA Donaldson C Denning-Kendall P Rice C Bradley B Hows JM Adult bone marrow is a rich source of human mesenchymal ‘stem’ cells but umbilical cord and mobilized adult blood are not. Br J Haematol (2003) 121(2):368–74 10.1046/j.1365-2141.2003.04284.x 12694261

[15] Alm JJ Koivu HM Heino TJ Hentunen TA Laitinen S Aro HT Circulating plastic adherent mesenchymal stem cells in aged hip fracture patients. J Orthop Res (2010) 28(12):1634–42 10.1002/jor.21167 20540091

[16] Rochefort GY Delorme B Lopez A Herault O Bonnet P Charbord P Multipotential mesenchymal stem cells are mobilized into peripheral blood by hypoxia. Stem Cells (2006) 24:2202–8 10.1634/stemcells.2006-0164 16778152

[17] Chen Y Xiang LX Shao JZ Pan RL Wang YX Dong XJ Recruitment of endogenous bone marrow mesenchymal stem cells towards injured liver. J Cell Mol Med (2010) 14(6B):1494–508 10.1111/j.1582-4934.2009.00912.x 19780871 PMC3829016

[18] Gil-Ortega M Garidou L Barreau C Maumus M Breasson L Tavernier G Native adipose stromal cells egress from adipose tissue in vivo: evidence during lymph node activation. Stem Cells (2013) 31(7):1309–20 10.1002/stem.1375 23533182

[19] Hoogduijn MJ Crop MJ Peeters AM Korevaar SS Eijken M Drabbels JJ Donor-derived mesenchymal stem cells remain present and functional in the transplanted human heart. Am J Transplant (2009) 9(1):222–30 10.1111/j.1600-6143.2008.02450.x 18976299

[20] Lama VN Smith L Badri L Flint A Andrei AC Murray S Evidence for tissue-resident mesenchymal stem cells in human adult lung from studies of transplanted allografts. J Clin Invest (2007) 117(4):989–96 10.1172/JCI29713 17347686 PMC1810571

[21] Jin SZ Liu BR Xu J Gao FL Hu ZJ Wang XH Ex vivo-expanded bone marrow stem cells home to the liver and ameliorate functional recovery in a mouse model of acute hepatic injury. Hepatobiliary Pancreat Dis Int (2012) 11(1):66–73 10.1016/S1499-3872(11)60127-6 22251472

[22] Jackson JS Golding JP Chapon C Jones WA Bhakoo KK Homing of stem cells to sites of inflammatory brain injury after intracerebral and intravenous administration: a longitudinal imaging study. Stem Cell Res Ther (2010) 1(2):17 10.1186/scrt17 20550687 PMC2905093

[23] Assis AC Carvalho JL Jacoby BA Ferreira RL Castanheira P Diniz SO Time-dependent migration of systemically delivered bone marrow mesenchymal stem cells to the infarcted heart. Cell Transplant (2010) 19(2):219–30 10.3727/096368909X479677 19906330

[24] Herrera MB Bussolati B Bruno S Morando L Mauriello-Romanazzi G Sanavio F Exogenous mesenchymal stem cells localize to the kidney by means of CD44 following acute tubular injury. Kidney Int (2007) 72(4):430–41 10.1038/sj.ki.5002334 17507906

[25] Liu N Patzak A Zhang J CXCR4-overexpressing bone marrow-derived mesenchymal stem cells improve repair of acute kidney injury. Am J Physiol Renal Physiol (2013) 305(7):F1064–73 10.1152/ajprenal.00178.2013 23884141

[26] Barbash IM Chouraqui P Baron J Feinberg MS Etzion S Tessone A Systemic delivery of bone marrow-derived mesenchymal stem cells to the infarcted myocardium: feasibility, cell migration, and body distribution. Circulation (2003) 108(7):863–8 10.1161/01.CIR.0000084828.50310.6A 12900340

[27] Kraitchman DL Tatsumi M Gilson WD Ishimori T Kedziorek D Walczak P Dynamic imaging of allogeneic mesenchymal stem cells trafficking to myocardial infarction. Circulation (2005) 112(10):1451–61 10.1161/CIRCULATIONAHA.105.537480 16129797 PMC1456731

[28] Lu W Fu C Song L Yao Y Zhang X Chen Z Exposure to supernatants of macrophages that phagocytized dead mesenchymal stem cells improves hypoxic cardiomyocytes survival. Int J Cardiol (2013) 165(2):333–40 10.1016/j.ijcard.2012.03.088 22475845

[29] Kunter U Rong S Djuric Z Boor P Muller-Newen G Yu D Transplanted mesenchymal stem cells accelerate glomerular healing in experimental glomerulonephritis. J Am Soc Nephrol (2006) 17(8):2202–12 10.1681/ASN.2005080815 16790513

[30] Zhong Y Tang Z Xu R Lin N Deng M Fang H Effect of transplantation route on stem cell migration to fibrotic liver of rats via cellular magnetic resonance imaging. Cytotherapy (2013) 15(10):1266–74 10.1016/j.jcyt.2013.05.023 23993301

[31] Amado LC Saliaris AP Schuleri KH St John M Xie JS Cattaneo S Cardiac repair with intramyocardial injection of allogeneic mesenchymal stem cells after myocardial infarction. Proc Natl Acad Sci U S A (2005) 102(32):11474–9 10.1073/pnas.0504388102 16061805 PMC1183573

[32] Friedenstein AJ Gorskaja JF Kulagina NN Fibroblast precursors in normal and irradiated mouse hematopoietic organs. Exp Hematol (1976) 4(5):267–74 976387

[33] Maumus M Peyrafitte JA D’Angelo R Fournier-Wirth C Bouloumie A Casteilla L Native human adipose stromal cells: localization, morphology and phenotype. Int J Obes (Lond) (2011) 35(9):1141–53 10.1038/ijo.2010.269 21266947 PMC3172585

[34] Braun J Kurtz A Barutcu N Bodo J Thiel A Dong J Concerted regulation of CD34 and CD105 accompanies mesenchymal stromal cell derivation from human adventitial stromal cell. Stem Cells Dev (2013) 22(5):815–27 10.1089/scd.2012.0263 23072708

[35] Colter DC Class R DiGirolamo CM Prockop DJ Rapid expansion of recycling stem cells in cultures of plastic-adherent cells from human bone marrow. Proc Natl Acad Sci U S A (2000) 97(7):3213–8 10.1073/pnas.97.7.3213 10725391 PMC16218

[36] Crop MJ Baan CC Korevaar SS Ijzermans JN Pescatori M Stubbs AP Inflammatory conditions affect gene expression and function of human adipose tissue-derived mesenchymal stem cells. Clin Exp Immunol (2010) 162(3):474–86 10.1111/j.1365-2249.2010.04256.x 20846162 PMC3026550

[37] Schrepfer S Deuse T Reichenspurner H Fischbein MP Robbins RC Pelletier MP Stem cell transplantation: the lung barrier. Transplant Proc (2007) 39(2):573–6 10.1016/j.transproceed.2006.12.019 17362785

[38] Spaggiari GM Capobianco A Becchetti S Mingari MC Moretta L Mesenchymal stem cell-natural killer cell interactions: evidence that activated NK cells are capable of killing MSCs, whereas MSCs can inhibit IL-2-induced NK-cell proliferation. Blood (2006) 107(4):1484–90 10.1182/blood-2005-07-2775 16239427

[39] Crop MJ Korevaar SS de Kuiper R JN IJ van Besouw NM Baan CC Human mesenchymal stem cells are susceptible to lysis by CD8(+) T cells and NK cells. Cell Transplant (2011) 20(10):1547–59 10.3727/096368910X564076 21396164

[40] Friedenstein AJ Petrakova KV Kurolesova AI Frolova GP Heterotopic of bone marrow. Analysis of precursor cells for osteogenic and hematopoietic tissues. Transplantation (1968) 6(2):230–47 10.1097/00007890-196803000-00009 5654088

[41] Pittenger MF Mackay AM Beck SC Jaiswal RK Douglas R Mosca JD Multilineage potential of adult human mesenchymal stem cells. Science (1999) 284(5411):143–7 10.1126/science.284.5411.143 10102814

[42] Horwitz EM Prockop DJ Fitzpatrick LA Koo WW Gordon PL Neel M Transplantability and therapeutic effects of bone marrow-derived mesenchymal cells in children with osteogenesis imperfecta. Nat Med (1999) 5(3):309–13 10.1038/6529 10086387

[43] Otsuru S Gordon PL Shimono K Jethva R Marino R Phillips CL Transplanted bone marrow mononuclear cells and MSCs impart clinical benefit to children with osteogenesis imperfecta through different mechanisms. Blood (2012) 120(9):1933–41 10.1182/blood-2011-12-400085 22829629 PMC3433095

[44] Togel F Hu Z Weiss K Isaac J Lange C Westenfelder C Administered mesenchymal stem cells protect against ischemic acute renal failure through differentiation-independent mechanisms. Am J Physiol Renal Physiol (2005) 289(1):F31–42 10.1152/ajprenal.00007.2005 15713913

[45] Melief SM Schrama E Brugman MH Tiemessen MM Hoogduijn MJ Fibbe WE Multipotent stromal cells induce human regulatory T cells through a novel pathway involving skewing of monocytes toward anti-inflammatory macrophages. Stem Cells (2013) 31(9):1980–91 10.1002/stem.1432 23712682

[46] Engela AU Hoogduijn MJ Boer K Litjens NH Betjes MG Weimar W Human adipose-tissue derived mesenchymal stem cells induce functional de-novo regulatory T cells with methylated FOXP3 gene DNA. Clin Exp Immunol (2013) 173(2):343–54 10.1111/cei.12120 23607314 PMC3722934

[47] Ge W Jiang J Arp J Liu W Garcia B Wang H Regulatory T-cell generation and kidney allograft tolerance induced by mesenchymal stem cells associated with indoleamine 2,3-dioxygenase expression. Transplantation (2010) 90(12):1312–20 10.1097/TP.0b013e3181fed001 21042238

[48] Nemeth K Leelahavanichkul A Yuen PS Mayer B Parmelee A Doi K Bone marrow stromal cells attenuate sepsis via prostaglandin E(2)-dependent reprogramming of host macrophages to increase their interleukin-10 production. Nat Med (2009) 15(1):42–9 10.1038/nm.1905 19098906 PMC2706487

[49] Mantovani A Biswas SK Galdiero MR Sica A Locati M Macrophage plasticity and polarization in tissue repair and remodelling. J Pathol (2013) 229(2):176–85 10.1002/path.4133 23096265

[50] Ben-Mordechai T Holbova R Landa-Rouben N Harel-Adar T Feinberg MS Abd Elrahman I Macrophage subpopulations are essential for infarct repair with and without stem cell therapy. J Am Coll Cardiol (2013) 62(20):1890–901 10.1016/j.jacc.2013.07.057 23973704

[51] Hoogduijn MJ Roemeling-van Rhijn M Engela AU Korevaar SS Mensah FK Franquesa M Mesenchymal stem cells induce an inflammatory response after intravenous infusion. Stem Cells Dev (2013) 22:2825–35 10.1089/scd.2013.0193 23767885

